# A new tetraploid species of *Solanum* L. sect. *Solanum* (Solanaceae) from Tanzania

**DOI:** 10.3897/phytokeys.16.2884

**Published:** 2012-08-24

**Authors:** Mkabwa L. K. Manoko, Gerard M. van Weerden, Ronald G. van Berg, Celestina Mariani

**Affiliations:** 1 Department of Botany, University of Dar es Salaam, Box 35060, Dar es Salaam, Tanzania; 2 Experimental Garden and Genebank, IWWR, Radboud University Nijmegen, Toernooiveld 11, 6525 ED Nijmegen, The Netherlands; 3 Biosystematics Group, Wageningen University, Radix building, Droevendaalsesteeg 1, 6708 PB Wageningen, The Netherlands; 4 Department of Molecular Plant Physiology, IWWR, Radboud University Nijmegen, Huygens building, Heyendaalseweg 135, 6525 AJ Nijmegen, The Netherlands

**Keywords:** New species, *Solanum umalilaense*, *Solanum* sect. *Solanum*, Tanzania, tetraploid, Umalila

## Abstract

*Solanum umalilaense* Manoko **sp. nov.** (Solanaceae) is described from the Umalila area, in the southern highlands of Tanzania. Its novelty is supported with both morphological and AFLP data. Phenetic and phylogenetic analyses place *Solanum umalilaense* as a unique and well-supported taxon among tetraploid species of *Solanum* sect. *Solanum* from Africa. It can be distinguished from other African species by its extremely developed branching, each branch producing many multi-flowered inflorescences, flowers with short calyx lobes and its persistent, small, light yellowish brown fruits.

## Introduction

*Solanum* L. section *Solanum* is a pantropical group of about 55 species, most of which are found in the New World, and about 10 in Africa; the group forms part of the Morelloid clade of Bohs (2005). *Solanum nigrum* L. is the type species for the section, and of the genus. Species in the section *Solanum* are herbs, sometimes suffrutescent and occasionally small shrubs. They also have simple, unbranched, uniseriate, multicellular hairs with or without glandular heads. Their ploidy levels range from diploid to hexaploid. Species with branched or stellate hairs do not fall in this section as traditionally circumscribed (Gray 1968). In Africa, section *Solanum* species form one of the largest groups of leafy vegetables.

The most thorough account of the African species of the section *Solanum*, with good morphological descriptions and keys, was published by Edmonds and Chweya (1997). Their list of native and introduced members of the section found in Africa includes *Solanum americanum* Mill., *Solanum chenopodioides* Lam., *Solanum nigrum* L., *Solanum physalifolium* Rusby var. *nitidibaccatum* (Bitter) Edmonds, *Solanum retroflexum* Dunal, *Solanum sarrachoides* Sendtn., *Solanum scabrum* Mill., *Solanum villosum* Mill. and other taxa such as *Solanum florulentum* Bitter, *Solanum grossidentatum* A. Rich., *Solanum hirsutum* (Vahl) Dunal and *Solanum tarderemotum* Bitter, which may represent good species and need taxonomic revision. Delimitation of species based on morphological data alone is complicated by phenotypic plasticity, polyploidy, natural hybridization and discordant variation in this group (Edmonds and Chweya 1997). Jacoby (2003) and Olet (2004) studied accessions of some species of the section *Solanum* found in Africa using also a molecular approach (Amplified Fragment Length Polymorphism, AFLP). Jacoby found that 15 morphological characters reflected the genetic variation observed in the AFLP analysis, and Olet defined species of the section *Solanum* found in Uganda by using morphological characterization and AFLP on living accessions. Whereas eight species were defined by morphological characters, only five were recognized using AFLPs.

While studying accessions of *Solanum* section *Solanum* from Africa at the Radboud University Nijmegen, it became apparent that plants grown from seeds of accession A24750133 collected fromthe Umalila area in the southern highlands of Tanzania were strikingly different from other African accessions. Chromosome counts revealed that they had 48 chromosomes, in common with some other species of the section in Africa (Manoko 2007). The plants were temporarily designated as “species A” in Schippers (2004).

During a herbarium visit in Dar es Salaam, Tanzania, we found a specimen of the same taxon (*R.E. Gereau et al. 5084*, DSM) collected on the 14^th^ of November 1992 from Umalila forest reserve. Duplicate specimens were present in NHT, MO and K. Annotation labels on these specimens indicated that they were identified by W.G. D’Arcy as *Solanum* aff. *americanum* Mill. and by L.B. Mwasumbi (DSM) as *Solanum nigrum* L.

We also located an additional specimen collected as a weed in maize field in Uyole (Mbeya) in the DSM herbarium (*A.A. Mwambunga A.A.M.6*). Fieldwork (July 2010) in Tanzania enabled us to collect a number of accessions of this species and here we formally describe it. Voucher specimens have been deposited at the Experimental Garden and Genebank herbarium of the Radboud University Nijmegen, DSM and WAG.

## Taxonomic treatment

### Solanum umalilaense

Manoko sp. nov.

urn:lsid:ipni.org:names:77121698-1

http://species-id.net/wiki/Solanum_umalilaense

Figs 1 , 3

#### Diagnosis.

*Solanum umalilaense* can be distinguished from other African species of *Solanum* sect. *Solanum* by its extremely developed branching, each branch producing many multi-flowered inflorescences, flowers with short calyx lobes and producing persistent, small, light yellowish brown fruits.

#### Type.

**Tanzania.** Mbeya: Mbeya District, Umalila Forest Reserve, ca. 7 km W of Ruanda II on road to Izumbwe, 2 km SSE of Mbogo Mtn. main peak, 09°11'S, 33°18'E, 2180 m, on bushy south-facing slopes with scattered shrubs. 14 Nov 1992. *R.E. Gereau, D.K. Harder, C.J. & M.J. Kayombo 5084* (holotype, DSM!; isotypes: K, not seen, MO, not seen, NHT!).

#### Description.

Herb, up to ± 0.5 m, erect, predominantly with many erect or spreading branches from the base; stems dark purple, angular with well marked ridges, teeth on ridges very small, glabrescent to sub-glabrous with sparse, long, appressed, eglandular hairs. Leaf simple, petiolate, ovate to elliptic with wings running to petiole base, margins entire, apex acuminate to acute, base truncate; petiole 0.6–1.3 cm; leaf blade 1.8–2.8 × 0.1–1.4 cm, widest 1–1.7(-2.1) cm from apex. Inflorescences numerous, up to 100 per plant, consisting of simple, rarely forked cymes, 2–9(-11) flowered; peduncle (0.5-)1.1–3.2, erect; pedicels 5–7(-9) mm, pendent. Calyx pentagonal, 3–3.2(-4) mm diam., lobes short (±1 mm), broadly triangular, equal and adherent to mature berries; corolla stellate, 7–11 mm diam. white, margins occasionally tinged with purple, style protruding the anthers, straight or sometimes hooked with eglandular hairs at the base, 3.1–3.5(-4.2) mm; anthers equal, yellow, 1.8–2 mm, filaments 0.5–1 mm; fully hydrated pollen 24.6–26.3 µm diam. Berries globose, changing from green to dull yellowish green, at mature stage light yellowish brown, texture soft, persistent and aromatic, 3–4(-5) mm diam.; sclerotic granules present; seeds obovate, brownish, 9 to 22 per berry. 1.6–2.1 × 1.3–1.8 mm.

Chromosome counts on accession A24750133 using standard cytological methods and flow cytometry on root and leaf cells respectively, showed that *Solanum umalilaense* had 48 chromosomes (2n=4x=48). The same was found for related African species such as *Solanum florulentum*, *Solanum tarderemotum*, *Solanum hirsutum*, *Solanum grossidentatum* and *Solanum retroflexum*, indicating that all these species are tetraploid.

In phenetic and cladistic analyses of an AFLP (Vos et al. 1995) dataset of the tetraploid species from Africa, including *Solanum villosum*, *Solanum retroflexum*, *Solanum florulentum*, *Solanum tarderemotum*, *Solanum grossidentatum* and *Solanum hirsutum*, *Solanum umalilaense* had 100% jackknife support in the neighbour joining and maximum parsimony analyses (Manoko 2007). A NJ tree was constructed using 435 polymorphic AFLP loci generated from 102 tetraploid individuals. Here only results for 44 individuals are shown, representing about 50% of the tree (Fig. 2; Manoko 2007), with *Solanum umalilaense* indicated as “sp A”. Cluster I was made up of individuals morphologically similar but not identical (Manoko 2007). Part of the material conformed to *Solanum tarderemotum* forms A and B described by Olet (2004), and two remaining accessions were *Solanum florulentum*. Individuals that conformed to *Solanum tarderemotum* forms A and B in this cluster shared morphological characteristics not only with *Solanum florulentum*, but also with *Solanum tarderemotum* form C. Cluster II was composed of *Solanum retroflexum* and a taxon that we identified as *Solanum hirsutum* (Manoko 2007). Both taxa have ovate calyx lobes, covering only half of the fruit during development, but reflexed away from mature fruits. The globose fruits, turning from green to purple/black, remain with the pedicel on the plant and are not aromatic. Cluster III is composed only of individuals of accession A24750133, which we describe here as the new species *Solanum umalilaense* (Fig. 2).

**Figure 1.**
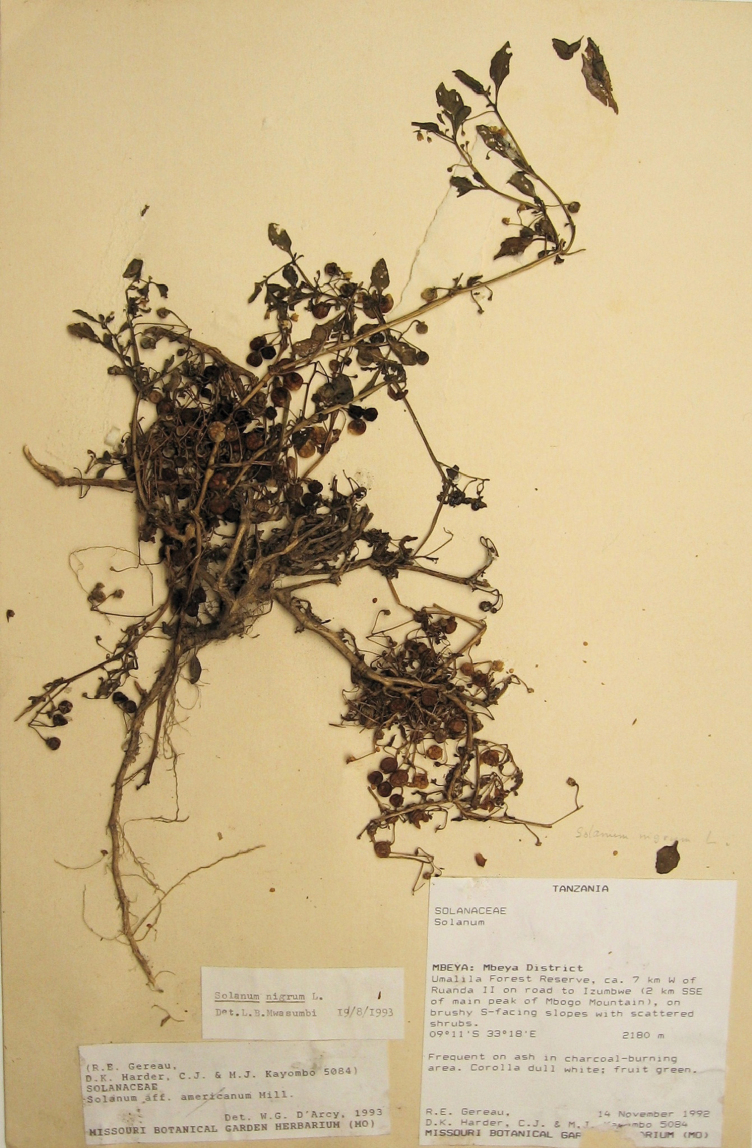
Holotype specimen of *Solanum umalilaense* Manoko sp. nov. (DSM)

**Figure 2.**
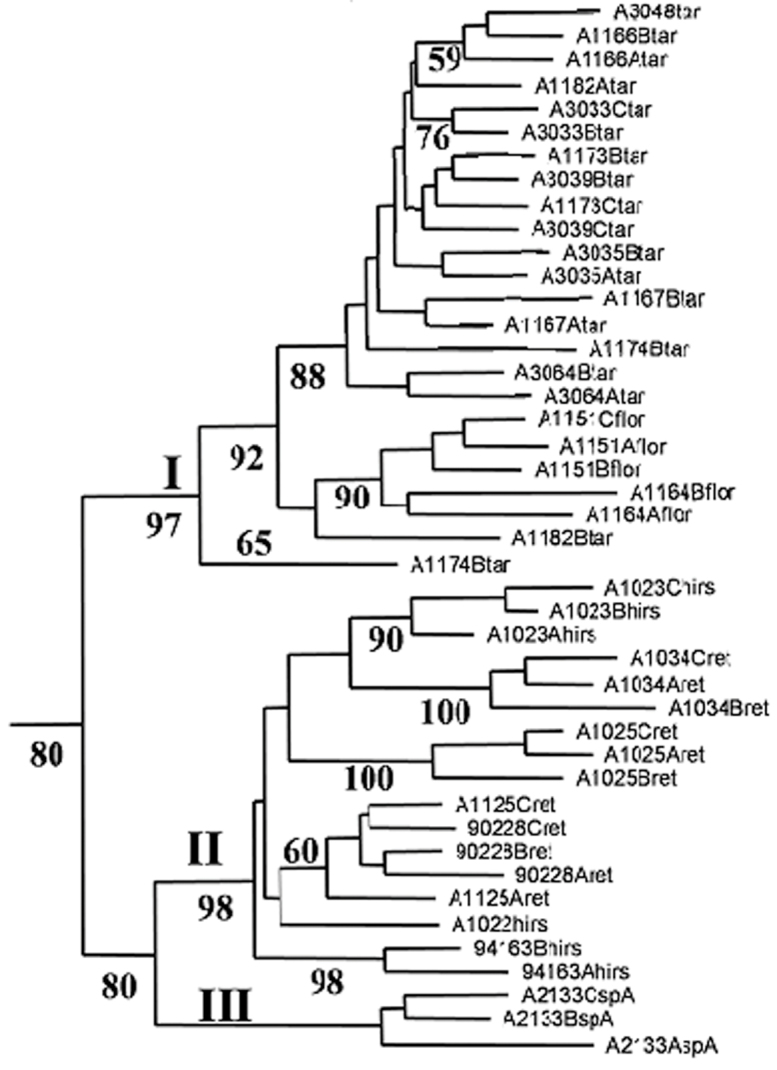
Part of the NJ tree from Manoko (2007). Numbers at the nodes are NJ Jackknife support values. Cluster I contains *Solanum tarderemotum* and *Solanum florulentum*. In cluster II are *Solanum retroflexum* and *Solanum hirsutum*. Cluster III is composed of individuals of *Solanum umalilaense*

#### Distribution.

Mbeya region, Tanzania, at elevations between 1952 and 2052 m (Fig. 4).

#### Ecology.

**Figure 3.**
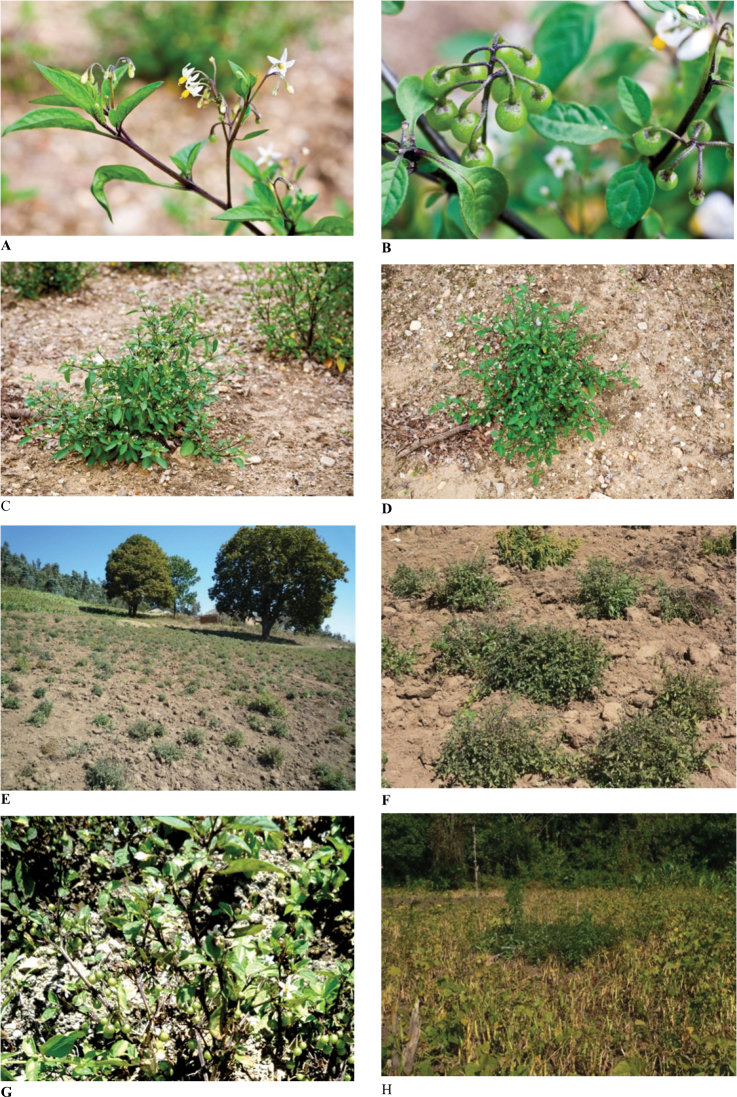
Pictures A,B,C,D taken from plants grown at the Experimental Garden, Radboud University Nijmegen (A24750133). Pictures E,F,G,H taken from plants growing in Mbeya region, Tanzania (E,F: Manoko et al. 2010-2; G,H: Manoko et al. 2010–14).

During our collecting trip in the Umalila area (July 7–10, 2010) we found many plants in cultivated fields (Fig. 3E,F) on the slope of mountains, or left in abandoned cultivated plots (Fig. 3H), which also contained maize and beans.

According to the information on *Gereau et al. 5084* the species is frequent on the ash layer in charcoal-burning areas. The Umalila area is located in the Mbeya region, in the South West of Tanzania at the border with Malawi and Zambia (Figure 3). Because of its elevation, the region is also known as the Southern Highlands of Tanzania, with volcanic type of soil, temperatures ranging from 12 to 23°C, and annual rainfall levels from 1500 to 2700 mm. The vegetation is mountainous, with cool temperature grasslands and the region is good for cultivation of coffee, maize, beans and vegetables (Anon. 1997).

#### Etymology.

The name *Solanum umalilaense* refers to the area the species was found. Umalila (Malilaor Umalila) is the highland area of the Malila, a relatively unknown ethnic group in south-west Tanzania (Walsh 1998).

#### Conservation status.

Based on the information on the type specimen, *Solanum umalilaense* grows in the Umalila forest reserve. We visited the villages of Maganjo, Isangati, Igala, and different wards and found that the species was cultivated as an important and well known leafy vegetable. It has been cultivated for many generations and farmers collect fruits and keep dried seeds for the next growing season. Since this species is cultivated as a food crop and has become almost domesticated, its preliminary conservation status can be considered to be of Least Concern (LC, IUCN 2001) although its very narrow distribution may be cause for further analysis.

#### Local names.

Insungwe, so called by the Malila (Schippers 2004).

#### Uses.

Used as a leafy vegetable. Leaves can be picked from the plant untill the plant starts flowering. Using leaves when the plant is in flower is not attractive because of their bitter taste (Latham 2006).

**Specimens examined. Tanzania. Mbeya Rural District:** Maganjo village (Lwindi ward), 09°02'29"S, 033°23'54"E, July 8, 2010, *Manoko et al. 2010–11* (DSM, WAG, and Radboud University Experimental Garden and Genebank herbarium); Isangati village (Iyunga ward), 09°04'12"S, 033°25'25"E, July 8, 2010, *Manoko et al. 2010-2* (DSM, WAG, and Radboud University Experimental Garden and Genebank herbarium); Igala village (Holondo ward), 09°03'21"S, 033°26'44"E, July 8, 2010, *Manoko et al. 2010–8* (DSM, WAG, Radboud University Experimental Garden and Genebank herbarium); Isangati village (Iyunga Mapinduzi ward), 09°05'03"S, 033°25'53"E, July 9, 2010, *Manoko et al. 2010–11* (DSM, WAG, Radboud University Experimental Garden and Genebank herbarium); Isangati village (Iyunga Mapinduzi ward), 09°05'03"S, 033°25'53"E, July 9, 2010, *Manoko et al. 2010–12* (DSM, WAG, Radboud University Experimental Garden and Genebank herbarium); field plot near Umalila Forest reserve, 09°11'26"S, 033°20'46"E, July 9, 2010, *Manoko et al. 2010–14* (DSM, WAG, Radboud University Experimental Garden and Genebank herbarium); Uyole (Mbeya), 5500 ft, 22 May 1968, *A.A. Mwambunga A.A.M.6* (DSM). **The Netherlands**. AccessionA24750133 from the Umalila area cultivated at Radboud University Experimental Garden and Genebank, *Anon. s.n.* (Radboud University Experimental Garden and Genebank herbarium).

#### Discussion.

*Solanum umalilaense* differs from all other species that have been described or studied so far from Africa (Bitter 1912; 1913; Bukenya and Hall 1988; D’Arcy and Rakotozafy 1994; Edmonds and Chweya 1997; Bosser et al. 2000; Jacoby 2003; Olet 2004; Gonçalves 2005; Edmonds 2006a, b). The new species produces a large number of inflorescences such that at full anthesis the plant appears to be covered with white flowers, strikingly different from other species in the section. Seeds of “species A”, as it was provisionally labeled, were received from the Southern Highlands of Tanzania where this taxon is a common leafy vegetable. Morphologically, it could not be assigned to any known species, therefore Schippers (2004) had given it the name species A. Herbaria surveys at NHT and DSM in Tanzania produced comparable material, all collected from Umalila Forest Reserve in Mbeya by R.E. Gereau et al. in 1992 (*R.E. Gereau, D.K. Harder, C.J. & M.J. Kayombo 5084*, the DSM specimen of which we designate as holotype). They had found these plants ‘frequent on ash, in charcoal-burning area’. During our collection-trip in Tanzania in the same region, however, we always found this species in cultivated plots, never in ash. Gereau’s material had been first determined by the late W.G. D’ Arcy (MO) in 1993 as *Solanum* aff. *americanum* Mill. Later, L.B. Mwasumbi (DSM) changed the name to *Solanum nigrum* L. These names refer to a diploid and to a hexaploid species, respectively, whereas *Solanum umalilaense* is tetraploid. This clearly demonstrates that the suggested affinities with *Solanum americanum* and *Solanum nigrum* were not correct.

Edmonds’s opinion (JM Edmonds pers. comm.) that it might be a hybrid between two tetraploid species was investigated. We found that for the three generations we grew the plants (between 2002–2004) none of the characters showed segregation.

All together, the distinct morphology, chromosome number different from *Solanum americanum* and *Solanum nigrum*, and a clear separate clustering from other species of the section, make this African species unique in section *Solanum* in Africa.

**Figure 4.**
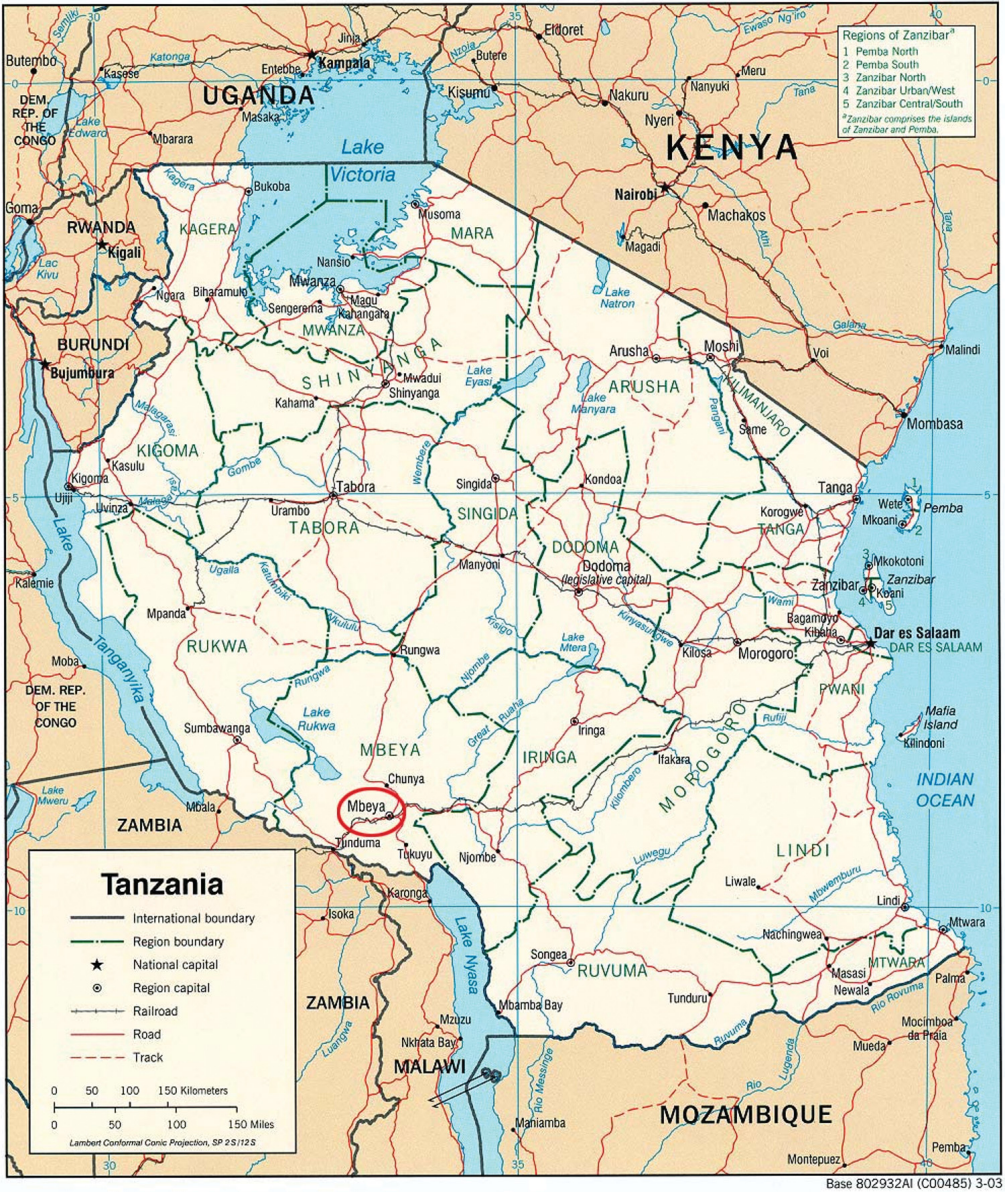
Map courtesy of the University of Texas Libraries, The University of Texas at Austin.

## Supplementary Material

XML Treatment for Solanum umalilaense
